# Acupuncture as an adjunctive therapy in a patient with diminished ovarian reserve and recurrent IVF failure: a case report

**DOI:** 10.3389/fmed.2026.1838061

**Published:** 2026-05-20

**Authors:** Zhihao Diao, Danyang Guo, Yiyang Sun, Chunjing Li, Na Zhang, Dongqing Du, Yuxia Ma

**Affiliations:** 1 Department of Acupuncture and Massage College, Shandong University of Traditional Chinese Medicine, Jinan, China; 2 The Second Affiliated Hospital of Shandong University of Traditional Chinese Medicine, Jinan, China; 3 Key Laboratory of Traditional Chinese Medicine Classical Theory, Ministry of Education, Shandong University of Traditional Chinese Medicine, Jinan, China

**Keywords:** acupuncture - therapy, DOR (diminished ovarian reserve), infertility, IVF (*in vitro* fertilization), TCM (trad. Chinese medicine)

## Abstract

**Background:**

Diminished ovarian reserve (DOR), characterized by reduced oocyte quantity and quality, presents clinically with menstrual disorders and hormonal disturbances, severely impacting fertility. The pathogenesis of DOR remains unclear and no treatment consensus currently exists. Acupuncture, a cost-effective and acceptable traditional Chinese medicine therapy, has gained widespread application in reproductive medicine. Emerging evidence indicates that acupuncture can improve ovarian function and enhance fertility potential in women with DOR.

**Case description:**

This article reports a case of a 38-year-old patient with DOR and recurrent *in vitro* fertilization (IVF) failure who achieved pregnancy following three months of acupuncture treatment, which was associated with improvements in sex hormonal levels, antral follicle count (AFC), and modified Kupperman Index (mKMI) score.

**Conclusion:**

This case report suggests that acupuncture as an adjunctive intervention may help improve ovarian function in patients with DOR and a history of recurrent failed IVF attempts, thereby providing evidence to support acupuncture as a potential treatment strategy for DOR-related infertility.

## 1 Introduction

Infertility is a major burden on global public health. According to World Health Organisation statistics, the prevalence of infertility among couples of reproductive age worldwide stands at 17.5% (1). Diminished ovarian reserve (DOR) constitutes a primary factor in female infertility (2). This age-related decline in ovarian function is characterized by a progressive reduction in both the quantity and quality of oocytes, leading to declining fertility. The characteristics of DOR are as follows: decreased antral follicle count (AFC), elevated basal follicle-stimulating hormone (FSH) levels, and reduced anti-Müllerian hormone (AMH) (3, 4). The diagnostic criteria are: (1) AMH < 1.1 ng/mL; (2) AFC < 5–7 (Days 2–4 of the menstrual cycle); (3) 10 IU/L ≤ basal FSH ≤ 25 IU/L (for two consecutive menstrual cycles) (5, 6). In severe cases, DOR can result in premature ovarian insufficiency, infertility and increasing risks of miscarriage and recurrent pregnancy loss (7, 8). Driven by socioeconomic development, lifestyle changes, and increasing work-related stress, the incidence of DOR has risen and affected a younger population. Epidemiological data indicates a significant increase in prevalence from 19% in 2004 to 26% in 2011 (9). Due to differences in diagnostic criteria, reported prevalence varies widely from 10 to 35%, while DOR accounts for approximately 20% among female ovarian disorders (8, 10–12). According to the 2022 national summary data on assisted reproductive technology (ART) published by the US Centers for Disease Control and Prevention (CDC), out of a total of 435,426 ART cycles, those undertaken due to DOR accounted for 26.2% of all ART cycles (13). Underscoring the clinical significance of this issue. Given its insidious and progressive profile, DOR may cause premature ovarian failure (POF) within 1 to 6 years (14). As DOR affects both quality of life and reproductive health, it poses a critical world-wide challenge in reproductive medicine, attracting research focus. Nevertheless, considerable controversy persists regarding its underlying causes and treatment approaches. Thus, exploring effective management strategies for DOR remains a paramount and ongoing challenge in clinical practice.

There is a lack of stably effective treatment for DOR, largely due to its heterogeneous clinical presentation (15). Current therapies antioxidant supplementation (e.g., coenzyme Q10, vitamins), hormone replacement therapy (HRT), dehydroepiandrosterone (DHEA) administration, growth hormone, ART, and intraovarian platelet-rich plasma (PRP) injections (14, 16–19). However, these therapeutic options only transiently improve hormonal levels and ovarian responsiveness. *In vitro* fertilization and embryo transfer (IVF-ET) outcomes remain suboptimal with low conception rates. Adverse effects of hormone use, long-period treatment cycles, and substantial costs further constrain the application (20, 21). Consequently, a growing number of women with DOR are seeking for complementary and alternative medicine, such as acupuncture, to enhance their therapeutic outcomes (22).

Acupuncture, a non-pharmacological intervention rooted in historical practices, is now widely applied in the field of reproductive medicine. Recent studies have witnessed the encouraging development of acupuncture for DOR, while the available evidences are limited and inconclusive. The mechanisms and therapeutic merits of acupuncture remain subjects of ongoing academic discussion (23). Acupuncture plays an important role of regulating endocrine function, promoting ovulation, modulating immune responses, and improving ovarian reserve and the local follicular microenvironment (24). Additionally, acupuncture has been associated with increased clinical pregnancy and live birth rates, thereby improving overall ART outcomes (25). This study reports on a case of acupuncture intervention in an advanced maternal age patient with DOR and a history of recurrent *in vitro* fertilization (IVF) failures, with the aim of assessing its clinical efficacy and providing clinical evidence for further research.

## 2 Case presentation

A 38-year-old female educator with a two-year history of primary infertility despite regular unprotected intercourse and multiple failed IVF attempts, for which she sought traditional Chinese medicine (TCM) treatment. The patient had a height of 170 cm, weight of 85 kg, and a body mass index (BMI) of 29.4 kg/m^2^. Her menarche occurred at the age of 13. Since 2024, the menstrual cycle had become irregular, characterized by a significant reduction in menstrual flow accompanied by blood clots. Laboratory tests (Fourth day of the period) indicated an AMH level of 0.11 ng/mL, luteinizing hormone (LH) of 3.62 mIU/mL, basal FSH of 11.61 mIU/mL (FSH/LH ratio approximately 3.21), estradiol (E2) of 97.58 pg./mL, and progesterone (P) of 2.04 ng/mL, revealing the possibility of DOR. Transvaginal ultrasound (TVS) demonstrated a total AFC of 5. She had unremarkable family history and normal chromosomal analysis, and the male partner’s semen analysis was within normal limits. Based on these findings, a diagnosis of DOR was established. Subsequently, the patient underwent five controlled ovarian stimulation cycles at a tertiary care hospital from 2024 to 2025, utilizing antagonist protocols, progestin-primed ovarian stimulation (PPOS), and natural cycle regimens. Each cycle yielded 1–2 oocytes, however, none developed into good-quality cleavage-stage embryos, leading to cancelation of all embryo transfer cycles.

**Figure 1:**
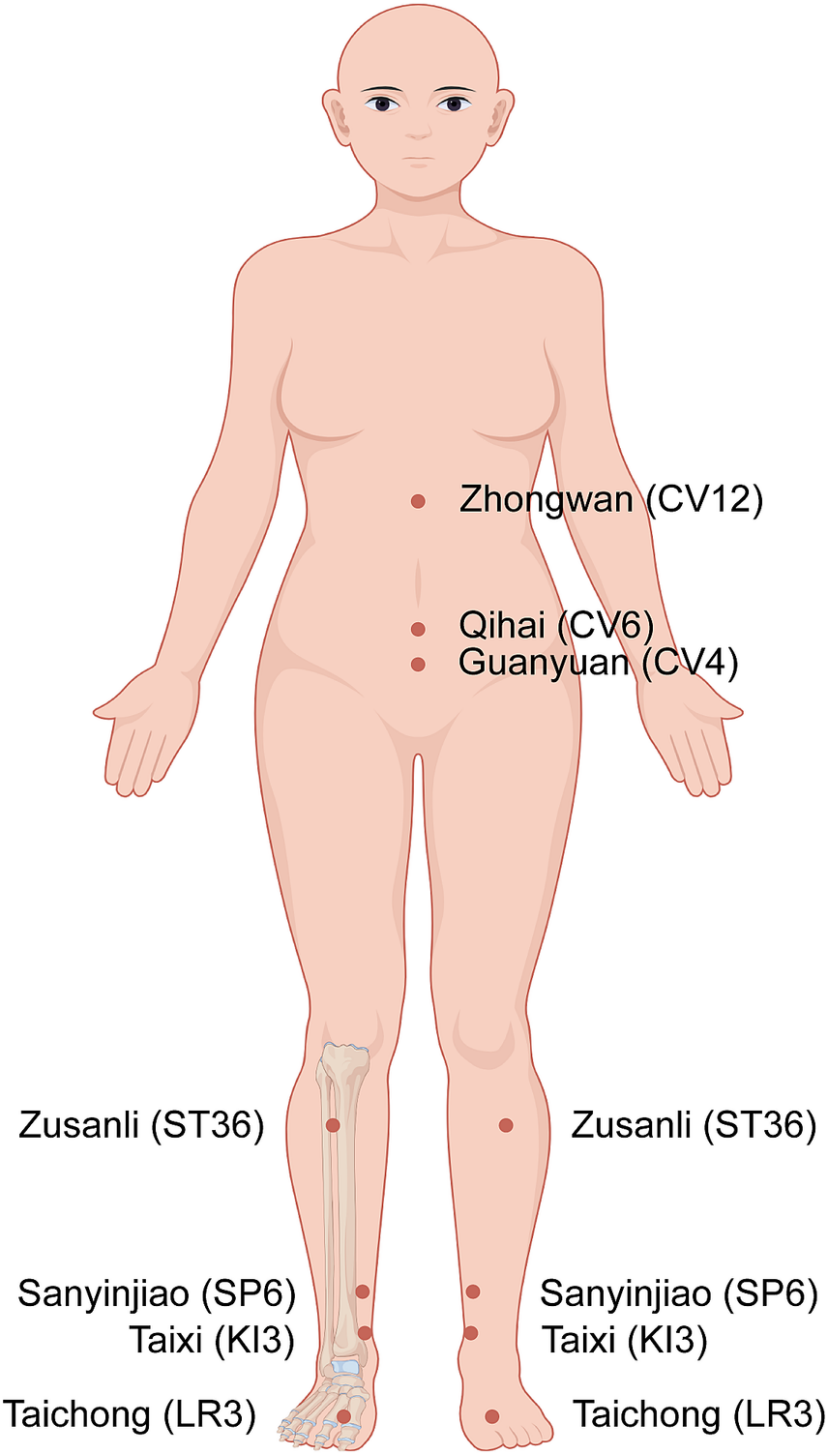
The location of acupoints. By Figdraw.

Prior to initiation, the patient underwent repeat hormonal assessment and transvaginal ultrasound on the fourth day in her menstrual cycle. The results suggested: FSH 22.83 mIU/mL, LH 6.12 mIU/mL, E2 23.93 pg./mL, P 0.53 ng/mL, endometrial thickness 4.5 mm, and AFC of 2 follicles in the right ovary (measuring 0.5 cm and 0.2 cm) and 0.2 cm follicle in the left ovary. Following the initial consultation, a comprehensive TCM diagnostic evaluation was performed by an experienced TCM practitioner utilizing the four diagnostic methods (inspection, auscultation and olfaction, inquiry, and palpation). The patient presented with a modified Kupperman Index (mKMI) score of 16, reflecting moderate menopausal symptoms including sensory disturbances, emotional irritability, fatigue, arthralgia, occasional insomnia, urinary symptoms, and dyspareunia. Combined with tongue and pulse findings, the acupuncturist formulated a standardized acupuncture protocol based on the TCM theory that “Conception Vessel Governs the uterus”. The treatment plan consisted of three sessions per week over three consecutive menstrual cycles, with acupuncture withheld during menstruation. Regular ovulation monitoring continued throughout the acupuncture treatment period. Acupuncture was administered by a licensed TCM practitioner with over three years of clinical acupuncture experience. Acupoints were localized strictly according to national acupoint location standards. The standardized acupuncture protocol included the following acupoints: Zhongwan (CV12), Guanyuan (CV4), Qihai (CV6), Sanyinjiao (SP6, bilateral), Zusanli (ST36, bilateral), Taixi (KI3, bilateral), and Taichong (LR3, bilateral) (Table 1; Figure 1). The patient was positioned supine to enable acupoints areas exposed. Following disinfection of both the skin and the sterile, disposable acupuncture needles (brand: Huatuo), the needles were inserted into corresponding acupoints. An even reinforcing-reducing manipulation technique was applied until the patient had a feeling of deqi sensation (a composite of sensations including soreness, numbness, distension, and heaviness) while maintaining tolerance. Needles were manipulated every 10 min and retained for 30 min per session.

**Table 1:** The location and depth of needle insertion at each point.

Acupoint	Standard location	Depth
Zhongwan (CV12)	On the upper abdomen, on the anterior midline, 4 cun superior to the umbilicus (CV8, Shenque).	25–40 mm
Qihai (CV6)	On the lower abdomen, on the anterior midline, 1.5 cun inferior to the umbilicus (CV8, Shenque)	25–50 mm
Guanyuan (CV4)	On the lower abdomen, on the anterior midline, 3 cun inferior to the umbilicus (CV8, Shenque).	25–50 mm
Zusanli (ST36, bilateral)	On the lower leg, on the anterior aspect of the leg, on the line connecting the laterosuperior border of the patella with the lateromalleolar prominence (ST35 with ST41), 3 cun inferior to ST35 (Dúbí), one finger-breadth (middle finger) lateral to the anterior crest of the tibia	25–50 mm
Sanyinjiao (SP6, bilateral)	On the lower leg, on the medial aspect of the leg, posterior to the medial border of the tibia, 3 cun superior to the prominence of the medial malleolus.	25–40 mm
Taixi (KI3, bilateral)	On the posteromedial aspect of the ankle, in the depression between the prominence of the medial malleolus and the calcaneal tendon.	15–25 mm
Taichong (LR3, bilateral)	On the dorsum of the foot, in the depression distal to the junction of the first and second metatarsal bones.	15–25 mm

**Figure 2:**
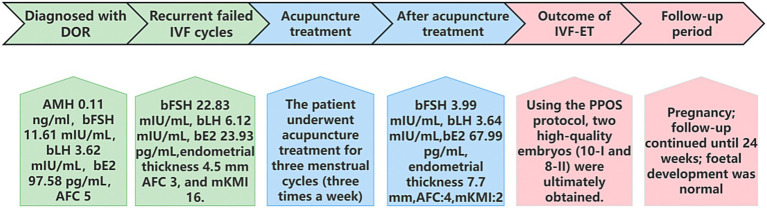
Patient treatment schedule.

Following completion of the three-month acupuncture treatment, the laboratory assessment on the fourth day in menstrual cycle demonstrated improved ovarian function: FSH 3.99 mIU/mL, LH 3.64 mIU/mL, E2 67.99 pg/mL, P 0.31 ng/mL, endometrial thickness 7.7 mm. Follicular development was observed, with the right ovary containing a 0.5 cm follicle and one additional small follicle, and the left ovary containing a 0.8 cm follicle and one additional small follicle. The mKMI score decreased to 2 (Table 2). Subsequently, a reproductive specialist initiated controlled ovarian stimulation using the PPOS protocol. On the nineth day in menstrual cycle, endometrial thickness was 9.0 mm, with follicular development as follows: right ovary: 2.0 cm and 0.7 cm follicles; left ovary: 1.5 cm and 0.35 cm follicles. Hormonal assessment revealed: LH 5.66 mIU/mL, E2 434.47 pg/mL, P 0.26 ng/mL. In this cycle, two oocytes were retrieved, both of which underwent normal fertilization and subsequently developed into two good-quality cleavage-stage embryos (one 10-cell grade I embryo and one 8-cell grade II embryo). Due to the use of the PPOS protocol, fresh embryo transfer was not performed. Instead, following endometrial preparation during a subsequent natural cycle, a frozen–thawed embryo transfer was conducted. Fourteen days post-transfer, serum *β*-hCG testing measured 845.45 mIU/mL, confirming biochemical pregnancy. As of the most recent follow-up at 24 weeks of gestation, nuchal translucency (NT) ultrasound, non-invasive prenatal testing (NIPT), and fetal anomaly scan have all revealed no abnormalities. No adverse events were observed during the treatment period. The schedule of intervention measures and specific treatment effects is shown in Figure 2.

**Table 2:** The patients’ baseline modified Kupperman Index (mKMI) scores, as well as their mKMI scores at 4, 8 and 12 weeks after treatment.

Symptoms	Weighting coefficient	Grades				Score			
		0	1	2	3	Base line	4 weeks after treatment	8weeksafter treatment	12weeksafter treatment
Sweating hot flushes	4	None	<3 times per day	3–9 times per day	>10 times per day	0	0	0	0
Paresthesia	2	None	Related to the weather	Feel tingling, burning, pricking, or numbness frequently	Loss of cold, warm and pain sensations	2	2	0	0
Insomnia	2	None	Occasionally	Often, need sleeping pill	Affects life and work	1	0	0	0
Nervousness	2	None	Occasionally	Often but controllable	Often and uncontrollable	1	1	1	0
Depression and suspicion	1	None	Occasionally	Often but controllable	Often and uncontrollable	0	0	0	0
Vertigo	1	None	Occasionally	Often but have no impact on life	Have impact on life and work	0	0	0	0
Fatigue	1	None	Occasionally	Feel difficult when climbing the 4th floor	Limited daily life	1	1	1	1
Arthralgia, myalgia	1	None	Occasionally	Often but functional normal	Dysfunction	1	1	1	1
Headache	1	None	Occasionally	Often but endurable	Often and need medication	0	0	0	0
Palpitation	1	None	Occasionally	Often but have no impact on work	Need to be treated	0	0	0	0
Formication	1	None	Occasionally	Often but endurable	Often and need medication	0	0	0	0
Urinary tract infection	2	None	Occasionally	More than 3 times per year, can heal itselfnot requiring medication	More than 3 times per year, needing medication	1	1	1	0
Sexual problems	2	Normal	Reduced libido	Dyspareunia	Loss of libido	2	0	0	0
Total						16	10	6	2

The symptom score is calculated as the product of the score and its weighting coefficient, while the total score represents the summation of all symptom scores.

## 3 Discussion

This case report describes a patient with advanced maternal age who was diagnosed with DOR and recurrent IVF failure. Baseline assessment demonstrated poor ovarian reserve indicators, including low AMH and AFC, elevated FSH, and an elevated FSH/LH ratio. The patient clinically presented with irregular menstrual cycles, emotional irritability, and insomnia. Following a three-month acupuncture intervention, improvements were observed across multiple aspects: ovarian function parameters and endometrial thickness showed enhancement, menstrual cycles became regular, and the mKMI score decreased. Notably, these improvements contributed into a favorable IVF-ET outcome, culminating in an ongoing pregnancy beyond 24 weeks of gestation with normal fetal development. Acupuncture may play a potential role in improving patients’ ovarian function and IVF-ET outcomes.

DOR remains one of the most challenging conundrums in reproductive medicine due to its multifactorial etiology and the absence of definitive curative therapies. Current treatment strategies for DOR primarily include ovulation induction medications, growth hormone, and DHEA supplementation, however, therapeutic responses are highly individualized, and a subset of patients exhibit poor responsiveness to these pharmacological interventions. In IVF cycles, patients with DOR frequently encounter challenges such as low oocyte yield, poor embryo quality, and elevated cycle cancelation rates (20, 21). Acupuncture, as a minimally invasive procedure and well-tolerated complementary and alternative medicine approach, demonstrated the potential to improve ovarian function parameters and pregnancy outcomes in this case. Consistent with findings from multiple previous clinical studies, acupuncture may exert its beneficial effects by reducing FSH levels, while concurrently increasing AMH levels and AFC, thereby optimizing the reproductive endocrine milieu. Acupuncture produces an effect through the multi-target regulation of the hypothalamic–pituitary-ovarian (HPO) axis (24, 26, 27). Furthermore, studies have found that acupuncture improves clinical pregnancy rates and live birth rates in women undergoing IVF-ET (28, 29). Acupuncture can enhance ovarian function through multifaceted mechanisms: regulating immune function, restoring ovarian tissue structure, improving microcirculation, promoting *β*-endorphin secretion, and inhibiting granulosa cell apoptosis (24, 30, 31).

It is worth noting that improvements in endometrial thickness were observed following acupuncture treatment. This enhancement created a more favorable environment for the subsequent frozen embryo transfer. Adequate endometrial receptivity is a critical determinant of successful embryo implantation. Although fresh embryo transfer was precluded by the use of the PPOS protocol, successful implantation was achieved during the subsequent frozen–thawed embryo transfer cycle, as confirmed by a serum *β*-hCG level of 845.45 mIU/mL. This suggests that acupuncture, in conjunction with subsequent endometrial preparation protocols, may have optimized endometrial receptivity. The underlying mechanisms may involve regulation of cytokine expression, reduction of uterine arterial blood flow resistance, and improvement of the endometrial microenvironment. The acupoint selection in this protocol was guided by the classic TCM theory that “the Conception Vessel governs the uterus” (Ren governs the Baotai), and focuses on improving indicators related to ovarian function, thereby offering a new potential approach to the treatment of DOR using acupuncture.

The inherent limitations of a single case report must be acknowledged. This case suggests that acupuncture may exert a certain modulatory effect on the reproductive function of advanced maternal age patients with DOR. However, given the limitations of a single-case study design, the above observations cannot rule out the potential influence of multiple confounding factors, such as the natural decline in ovarian function with age, changes in BMI, and individualized adjustments to IVF protocols. Consequently, further clarification requires prospective studies with more rigorous designs. It is important to note that follow-up to delivery has not yet been completed. Given that live birth is the key indicator for assessing the efficacy of interventions, this study has so far only followed participants up to 24 weeks’ gestation; the findings therefore do not yet reflect the final pregnancy outcome. Follow-up will be extended until delivery in order to further strengthen the evidence regarding the outcome.

## 4 Conclusion

In summary, this case study demonstrates that acupuncture, as an adjunctive treatment, has the potential to improve ovarian function in patients with DOR and recurrent IVF failure. Future research should prioritize well-designed, large-scale randomized controlled trials and basic research to validate the findings of this case and elucidate the molecular mechanisms by which acupuncture improves ovarian function and embryo quality.

## Data Availability

The original contributions presented in the study are included in the article/supplementary material, further inquiries can be directed to the corresponding authors.
